# A study protocol for interprofessional collaborative, digital, and sustainability training in primary healthcare: the REALISE study

**DOI:** 10.1017/S1463423625100455

**Published:** 2025-09-30

**Authors:** Marlene Brunner, Eva Maria Propst, Melanie Roth, Christine Kern, Joachim Schulze, Johanna Bodenhofer, Gertie Janneke Oostingh, Daniela Huber

**Affiliations:** Department for Health Sciences, Salzburg University of Applied Sciences, Salzburg, Austria

**Keywords:** Healthcare professionals, interprofessional collaboration, interprofessional education, primary healthcare, social professions, simulation, sustainability

## Abstract

**Background:**

Primary healthcare units (PHCUs) in Austria play a crucial role in providing regionally tailored, high-quality care through interprofessional teams. Barriers, such as limited training and unclear roles, hinder effective interprofessional collaboration (IPC). Additionally, healthcare and social professionals (HCSPs) in primary healthcare (PHC) face a rise in patients with non-communicable diseases and increasing climate-related challenges, underscoring the need for education addressing IPC and sustainability to build resilient healthcare.

**Aim:**

This paper presents the protocol of the REALISE study, which aims to evaluate the effectiveness of a didactic concept integrating collaborative, digital, and sustainability skills within multimodal training modules (including simulations).

**Methods:**

In this prospective trial, HCSPs working in PHC and students in their final year of education in related professions are recruited to participate in interprofessional training modules, which take place on four days within a month in person and with additional e-learning elements between those days. The modules consist of didactic elements on IPC and sustainability, simulation scenarios with acting patients, and immersive virtual reality scenarios. The primary outcomes assess IPC by utilizing the Teamwork Assessment Scale, the Interprofessional Socialization and Valuing Scale (9a/9b), and the Interprofessional Collaborative Competency Attainment Survey. Secondary outcomes focus on sustainability and environmental awareness, as well as the organization and structure of the training modules.

**Discussion:**

The findings of this study will demonstrate the effect of proprietary training modules on IPC and will inform on the integration of respective modules into standard curricula and continuing educational programmes at the Salzburg University of Applied Sciences.

## Introduction/Background

### Background

Austrian healthcare, like the global trend, is undergoing significant change, with the rise of non-communicable diseases (NCDs) posing a major challenge (GBD 2019 Diseases and Injuries Collaborators, 2020). Addressing NCDs requires interprofessional collaboration (IPC) at community level, which is primarily centred in the field of primary care (Van Weel and Kidd, 2018). This is one of the key reasons why strengthening the primary healthcare (PHC) sector and primary healthcare units (PHCUs) is a major focus in the ongoing reform of Austria’s healthcare. The aim is to relieve the burden on the expensive hospital sector and provide patients with high-quality healthcare close to home (Bundesministerium Soziales, Gesundheit, Pflege und Konsumentenschutz, 2024). Expanding and improving the legal framework for primary care facilities has therefore been a political focus. A revision of the Primary Care Act also aimed to enhance the appeal of PHC to healthcare and social professionals (HCSPs) by offering enhanced opportunities and appealing working conditions (Parlamentsdirektion, 2023). At the start of 2025, Austria has 83 PHCUs (Plattform Primärversorgung, 2025), and there are efforts to further expand PHCUs throughout Austria (Bundeskanzleramt Österreich, 2023).

PHCUs in Austria offer continuous medical care and coordination of further health services, and they are realized in two organizational forms: in PHC networks, where the team is dispersed over the service region, and in PHC centres, where the interprofessional team is situated at one location (*Bundesgesetz über die Primärversorgung in Primärversorgungseinheiten (Primärversorgungsgesetz – PrimVG).* § 2 BGBl. I Nr. 131/2017, 2023). PHCUs consist of three required professions (general practitioners, nurses, and medical office assistants) and other healthcare and social professions depending on the demand in the region (speech and language therapists, physiotherapists, occupational therapists, dieticians, midwives, social workers, psychotherapists, health-psychologists, clinical-psychologists, PHCU managers). In PHCUs specialized on children, paediatricians are part of the team instead of or in addition to general practitioners (Plattform Primärversorgung, 2022). These professional groups (PG) combine their unique competencies and skills to work towards common goals and address complex patient problems (Rawlinson *et al.*, 2021).

IPC has shown a significant positive influence on patient outcomes (Tandan *et al.*, 2024) and patient safety (Purnasiwi and Jenie, 2021). Problems faced by HCSPs in primary care include physical distance, fears about professional identity, poor communication, and a lack of training and clear roles as barriers to successful IPC (Rawlinson *et al.*, 2021). To counteract these problems, IPC in practice requires interprofessional teaching, research, and an active link between teaching, learning, and practice (Guraya and Barr, 2018). Future HCSPs undergo training programmes of varying durations and levels, provided by different and physically separate educational institutions (Federal Ministry of Social Affairs, 2020). Therefore, some HCSPs have their first contact with other professions only during their internships or even after graduation.

Furthermore, HCSPs encounter various issues related to planetary health in their daily patient interactions. For example, rising temperatures lead to more cardio-pulmonary challenges (Copernicus, 2023; Luterbacher *et al.*, 2004; R. Twardosz *et al.*, 2021) as well as an increased prevalence of allergies and respiratory diseases (M. Blaiss, 2020; Traidl-Hoffmann, 2017). In contrast to these problems, the existing climate awareness among healthcare professionals remains inadequately translated into actions (Rangel *et al.*, 2024). Furthermore, the healthcare sector itself currently accounts for 7% of CO_2_ emissions in Austria (Weisz *et al.*, 2020). Consequently, further training is to be provided to employees of healthcare facilities on both climate protection and climate change adaptation (Lichtenecker *et al.*, 2024). Addressing these environmental and health challenges requires changes within the healthcare sector. In this context, the WHO framework for resilient and sustainable health systems prioritizes PHC as an anchor for health systems and the strengthening of community-based health-related interventions (WHO Regional Office for Europe, 2024).

In summary, there is a need for evidence-based didactic concepts that strengthen interprofessional and sustainable cooperation of HCSPs in primary care in basic as well as in continued education. Simulation-based training modules could be an appropriate teaching method to achieve these goals (Sezgin and Bektas, 2023).

### Objectives and trial design

This paper presents the study protocol of the REALISE study (‘Collaborative, Digital and Sustainable Skills through Multimodal Simulation: Evidence-Based, Interprofessional Training in Primary Healthcare’), a prospective trial with the objective of evaluating the impact of interprofessional training modules in a PHC setting as an intervention for interdisciplinary collaboration based on the SPIRIT-guidelines for clinical trial protocols (Chan *et al.*, 2013). This study will answer the following two questions: How does the integration of collaborative, digital, and sustainability skills in multimodal training modules influence the development of interprofessional competencies in primary health and social care education? And what impact do these training modules have on the implementation of green and sustainable practices in health and social care?

## Materials and methods

### Participants, interventions, and outcomes

#### Study setting

The REALISE study is developed as a prospective trial to investigate interprofessional, complex, and multimodal training modules for HCSPs and students. The study design involves the implementation of three separate four-week learning paths over parallel periods. This study will serve as a feasibility study for further implementation. Each learning path will consist of four days of attendance at the Salzburg University of Applied Sciences supplemented by blended learning elements. This will be followed by a two-month follow-up phase.

#### Eligibility criteria

The REALISE study will be open to people aged 18–68 years, who are currently employed or studying in one of the 16 defined PG (general medicine, healthcare and nursing, orthoptics, biomedical analysis, dietetics, midwifery, speech therapy, medical training therapy, pharmacology, physiotherapy, occupational therapy, surgery assistance, psychology, radiology technology, social work, and paediatrics). Experienced HCSPs will be included if they work in primary care together with other health or social care professionals and have regular patient contact. They must have at least two years’ experience in primary care or related fields. Students are only included if they are in their final year of training/studies in their respective professions. People of all genders and backgrounds are included. An informed consent must be signed by each participant. To facilitate the team-building process, prospective participants who are unable to commit to the entire learning path will be excluded.

#### Interventions

The learning paths are developed for interprofessional groups of seven to ten people, consisting of a mix of students and HCSPs. Six case scenarios with a focus on primary health and social care, two per learning path, are created as the main component of this study. These include simulations with actor patients and immersive virtual reality (iVR) environments. Parallel online contents, such as presentations, videos, and assignments, are created, which are going to be supplemented by additional didactic elements in person, and a digital communication tool (Medikit) is included close before learning paths start. To ensure high quality and professional correctness, the case scenarios are revised and adapted in close collaboration with project partners and representatives of various PGs from primary care. Case scenarios are based on fictitious but realistic patient cases that are specifically designed to require intensive IPC and communication. These scenarios are grounded in the following eleven areas of PHC proposed by the Austrian Structural Plan for Health as a guideline: health promotion and prevention, family planning, pregnancy counselling, and infant examinations, basic outpatient care and follow-up of acute cases, acute care and follow-up of complex cases, rehabilitative therapy, long-term care for chronically ill and multimorbid patients, special care requirements for children and adolescents, special care requirements for the elderly, special care requirements for individuals with mental illnesses, and palliative care (Federal Ministry of Social Affairs, Health, Care and Consumer Protection, 2019). The scenarios are built around the following patient examples, which were focused on NCDs, which also consider the influence of climate change on these clinical cases: a geriatric couple with internal problems, a young mother after a stroke, a widowed elderly woman with pain symptoms, a young male athlete with long COVID symptoms, a family man with migrant background and diabetes mellitus type II and a pregnant woman with pre-eclampsia. The schematic schedule applicable to all learning paths can be found in Table 1. The entire REALISE study is planned to be conducted at the Salzburg University of Applied Science in Puch/Urstein (Austria, 47.727128° N, 13.085423° E).

**Table 1.**
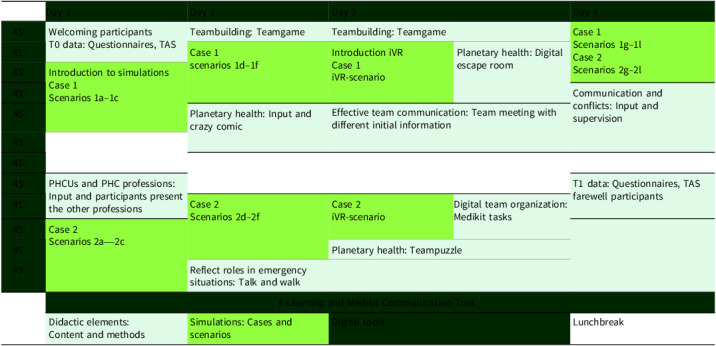
Thematic schedule of interventions

*Note:* TAS = Teamwork Assessment Scale; PHCUs = primary healthcare units; PHC = primary healthcare; iVR = immersive virtual reality.

##### Simulation training with acting patients and high-fidelity manikins

Simulation-based training has gained prominence in healthcare and social work education, offering participants risk-free opportunities to practice skills and develop competencies (Asakura *et al.*, 2021; Sollars and Xenakis, 2021). Various simulation methods, including acting patients (APs) and high-fidelity manikins, have already been used to enhance interprofessional education (Nimmagadda and Murphy, 2014).

The simulation trainings for the three learning paths of the REALISE study were developed based on the TeamSTEPPS ® Concept 3.0 (Parker *et al.*, 2019) with the following three focal points: improving patient and staff safety, improving communication and teamwork skills among HCSPs, and improving efficiency within patient care. In the course of the investigation, the focus on patient safety will be trained through targeted crew resource management (CRM) according to Rall (Rall, 2013). The 15 guiding principles of CRM and the importance of human factors, which play a decisive role in emergency care, will be taught in detail in a theoretical unit (Rall, 2013).

When planning the simulation training, care is taken to integrate the relevant PGs on a case-by-case basis. Great importance is attached to tailoring the prerequisites and learning objectives (cognitive, affective, and psychomotor) to the specific requirements of the PGs involved as early as the case construction stage and during operationalization. A set of expectations is developed in advance for each simulation, considering the respective requirements and learning objectives. During the planned study, all simulations will be performed with APs; in the case of an emergency simulation, high-fidelity manikins will be used for invasive techniques. In the implementation phase, a briefing for each scenario is planned to clearly and comprehensibly explain the settings and the scenario overview to the APs and participants.

Each scenario will be conducted according to the principles of simulation pedagogy (Jeffries, 2012), taking into account Kolb’s learning cycle (St. Pierre and Breuer, 2013). Particular care will be taken to establish and maintain a psychologically safe learning environment for all participants (Turner and Harder, 2018). Usually, three different roles will be performed by APs in every scenario– mostly patients, relatives, or practice assistants. The nature of the roles will depend on the specific case and scenario, as well as on which disciplines the interprofessional team will be able to cover on its own and which additional role(-s) will be needed (e.g. paramedics). The purpose of this will be to provoke the defined learning objectives, which will then be discussed in the debriefing. The debriefing will be conducted according to the principles of Gaba and Dieckmann (Dieckmann, 2018).

##### Virtual reality simulation training

Virtual reality (VR) is emerging as a valuable tool for HCSP education and offers numerous complements to traditional methods. It provides an interactive, engaging learning environment that supports experimental learning by doing in a highly standardizable and therefore repeatable setting, which could improve knowledge and skill transfer (Kyaw *et al.*, 2019; Manolakis and Papagiannakis, 2022). VR applications in health and social care can cover a wide range of professional training, such as potential applications in various disease prevention and management settings (Aziz, 2018). Recent research has focused on the use of VR for training non-technical skills, such as teamwork, communication, and situational awareness (Bracq et al., 2019).

To supplement the simulations with APs in the REALISE study, a continuation of the case scenarios’ content will be realized in an iVR environment, in which participants are actively part of the simulation themselves by means of an avatar, for one of the four attendance days. This will be carried out by creating and using especially tailor-made and newly developed immersive scenarios with patient examples in the iVR software UbiSim V1.16 by Labster (Montreal, Canada; https://www.ubisimvr.com/). This tool provides iVR training and simulation for healthcare, offering a high-fidelity simulation experience where clinical judgement can be practised within a realistic setting.

##### Didactic elements

The didactic elements are developed in line with the level of university education (Rhein and Wildt, 2023), aiming to deepen IPC as well as sustainable and environmentally conscious work in PHC. For example, team-building exercises, fictitious case discussions in a team meeting, reflections in individual and group settings, mutual case presentations, and presentations of one’s own and other PGs, as well as an intensive focus on communication skills, will be applied.

##### Blended learning and Medikit communication tool

E-learning has become increasingly important in health and social science education, as it provides flexible access to learning resources and it can be as effective as traditional methods in many areas of health and social professional education (Lawn *et al.*, 2017; Vaona *et al.*, 2018). Successful implementation of e-learning requires consideration of learner motivation, user-friendly technology, and learner-centred pedagogy (Regmi and Jones, 2020). Blended learning approaches that combine e-learning with face-to-face methods could be particularly effective in developing complex skills such as supporting self-management (Lawn *et al.*, 2017). In interprofessional education, both students and HCSPs perceive the use of information and communication technologies positively (Curran *et al.*, 2015). In addition to, or as individual preparation and follow-up to the subject-specific content that will be carried out during the face-to-face phases of the intervention, various – partly interactive – learning contents will be offered via a Moodle platform (Dublin, Ireland; https://moodle.com/ ). Materials such as theoretical background information on clinical patterns of the ‘patients’ in the case scenarios, videos, and links on the topic of sustainable and environmentally conscious work, as well as content on the communication tool (Medikit), will be available for participants during learning paths.

The Medikit tool (hotelkit GmbH, Salzburg, Austria; https://medikit.net/de/ ), a digital platform for healthcare and social services, will be used for communication among the study participants and with the project team to collaborate independent of time and place. An escape room to facilitate team-based problem-solving on sustainability issues will also be embedded in the Moodle platform.

#### Outcomes

The data will be collected using a combination of self-assessments and observations. At the beginning (T0) and directly after the completion of the learning path (T1), the participants will answer an online questionnaire containing demographic information questions on the set up of the learning path (the time structure and the organization of the learning path) and validated instruments on IPC (Interprofessional Socialization and Valuing Scale 9a/9b (ISVS) (Gunaldo *et al.*, 2024), Interprofessional Collaborative Competency Attainment Survey (ICCAS) (Archibald *et al.*, 2014). In addition, the participants of each learning path will be given the same team task with a minimally modified challenge at the beginning (rope square) and end (rope triangle) of each learning path, which is specifically geared towards the requirements of communication and teamwork. This exercise will be videotaped and then evaluated by three independent, blinded, and specialized raters using the Teamwork Assessment Scale (TAS) (Kiesewetter and Fischer, 2015). In a follow-up, two months after completion of the learning path (T2), the working participants will be asked via an online survey whether and to what extent the learning paths have influenced their work practice. As part of the learning module feasibility survey, two months was deemed sufficient to gain initial insights and assess the feasibility of the training programme; furthermore, a shorter duration allows for faster data collection and analysis, which enables timely adjustments and improvements in any follow-up work from a scientific and higher education didactic perspective. This focus on a shorter follow-up period also reflects the study’s particular interest in short-term effects on participants’ professional practice. Furthermore, qualitative questions on the development of interprofessional competences of HCSP and students and on the implementation of green and sustainable practices in their own practical work will be conducted at all timepoints with the respective participants (see Table 2). The results of the validated instruments ISVS, ICCAS, and TAS are defined as primary outcomes, all others as secondary outcomes (see Table 2).

**Table 2.**
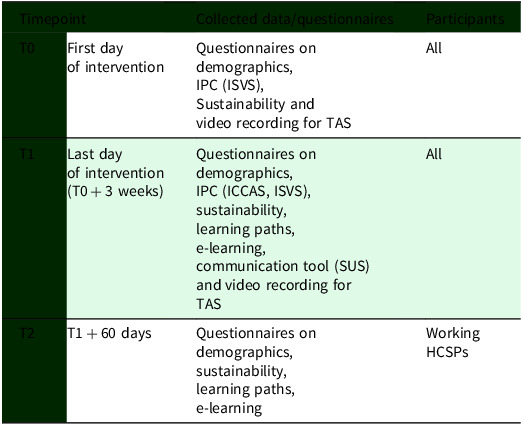
Data collection

*Note:* IPC = interprofessional collaboration; ISVS = Interprofessional Socialization and Valuing Scale; TAS = Teamwork Assessment Scale; ICCAS = Interprofessional Collaborative Competency Attainment Survey; SUS = System Usability Scale; HCSPs = healthcare and social professionals.

#### Participant timeline

**Figure 1.**
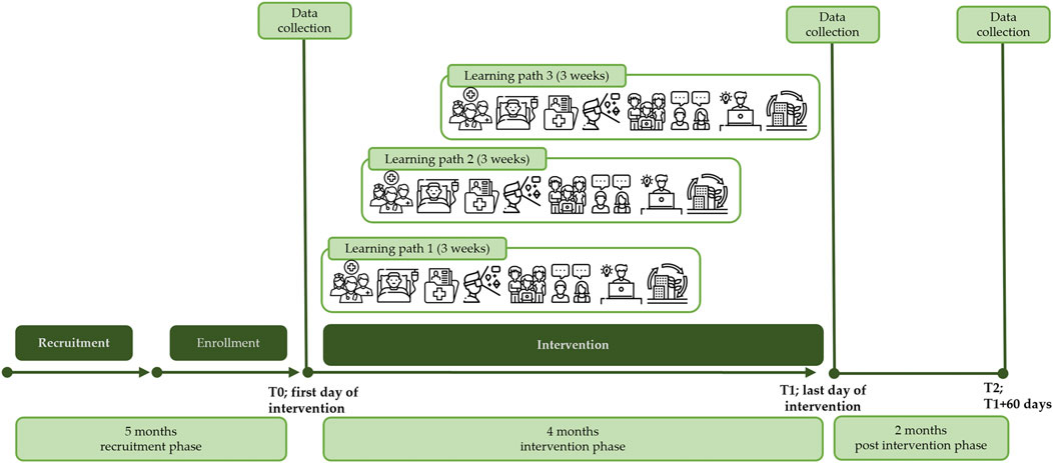
Participant timeline of enrolment, interventions, and assessments (Eucalyp, 2024a, 2024b; Freepik, 2024a, 2024b; Hajicon, 2024; JuicyFish, 2024; Kosonicon, 2024; RaftelDesign, 2024).

Three learning paths will be offered, in which HCSPs and students will work together as an interprofessional team on two patient cases including different activities. Each participant will take part in one learning path. In total, the participants will spend around 40 teaching units on their learning path. The four days of attendance will be spread out over three weeks. Day one marking T0 and day four marking T1. A chronological sequence of the study implementation can be found in Figure 1.

#### Sample size

To achieve the study objectives, the estimated number of participants is based on three different learning paths, each of which will have to be completed by 7–10 individuals. The number of participants is determined based on didactical considerations (Au *et al.*, 2023; Jeffries, 2012). Therefore, a total of around 21–30 participants are planned. A formal sample size calculation will not be carried out as this will be a feasibility study with the main objectives to gain insights into the learning process and the application of the simulations in different learning paths. For targeted recruiting, an allocation scheme regarding the content-related proximity of the PGs to the case scenarios in the respective learning path will be created; for each learning path, one place per PG will be provided for students and one for HCSPs. If a sufficient number of people are interested in participation, stratification by gender, age, origin, and professional experience will be carried out.

#### Recruitment

The recruitment of participants will be organized through a variety of communication channels. The plan is to directly approach stakeholder groups and relevant professional associations and task them with disseminating information about study participation. Furthermore, a targeted email campaign will be conducted to reach PHCUs, rehabilitation centres, and general practitioners’ offices in Salzburg and the surrounding regions. To further increase the visibility of the study, information flyers will be distributed at relevant events and scientific conferences. Digital communication platforms, such as the Austrian PHC platform, as well as social networks like LinkedIn, Facebook, and Instagram, will be strategically employed to reach a broad and diverse target audience. Additionally, requests will be made to the educational institutions for HCSPs in Salzburg and neighbouring federal states, encouraging their students to participate in the study.

### Data collection, management, and analysis

#### Data collection methods

All data for the evaluation will be digitally collected via online surveys. To avoid missing data, all questions in the individual online survey will be mandatory. If study participants opt to leave the study prematurely, their data will no longer be used for analysis. The entire data collection will be carried out by experienced researchers from the project team.

#### Data management

The collected data will be stored on the university’s internal servers, accessible only by selected members of the project team. All persons authorized to access the data are subject to the Austrian and European data protection law and are obliged to maintain data confidentiality. The participants’ data will be anonymized by means of a three-digit number ID; the master file for decoding this process will only be accessible to the study management in the context of study recruitment and participant withdrawal.

#### Statistical methods

Analysis of the primary outcomes will descriptively compare the participants’ change in IPC (via the ISVS 9A and 9B) to their retrospective perception of the impact of the training modules on IPC. These self-reported outcomes will be discussed in the light of the external observations made (via the TAS) at the corresponding timepoints. In detail, intragroup comparisons will be made using a paired t-test, Fisher exact test, or Wilcoxon test; comparisons between the groups will be made using an ART ANOVA (aligned rank transform analysis of variance) and a post-hoc test. The significance level will be set at *p* < 0.05. All statistical analyses will be performed with the R software from R Foundation (Vienna, Austria; https://www.r-project.org/) in accordance with the per-protocol principles. The study design of the publication and consequently the publication of the results will comply with the CONSORT Statement (Cuschieri, 2019).

#### Data monitoring

Throughout the REALISE study, the implementation is monitored by the study coordinators. The project team will ensure that all participants meet the inclusion criteria and that the study protocol is conducted as planned. Interim analysis will not be performed. A data monitoring committee will not be established. Adverse events, although the intervention is considered to pose a very low risk, will be monitored by the project team and will have to be registered and reported to the study coordinators and the institutional ethics committee of the [Anonymized].

### Ethics and dissemination

The study protocol of the REALISE study is approved by the institutional ethics committee of the [Anonymized] with reference number [Anonymized]. The statistical analysis will be based on anonymized data, and correspondingly, the findings will be published anonymously in all presentations at national and international conferences and publications in peer-reviewed journals. All documents and data related to the study will be saved in the electronic archives of Salzburg University of Applied Sciences for the required period of at least 10 years and can be viewed on reasonable requests after publication. The authors have no conflicts of interest.

## Discussion

IPC training modules, including simulations, have predominantly been designed for clinical settings. Within the REALISE study, they are now being adapted for the PHC context for the first time, focusing on the long-term care of patients with NCDs from a comprehensive, interprofessional, and sustainable health and social care perspective. This adjustment reflects the increasing recognition of the PHC’s importance in Austria and addresses the specific requirements of this growing sector. Including both HCSPs and students of the respective professions will offer valuable insights for curriculum integration and facilitate the refinement of the concept for implementation in continuing education programmes.

IPC training including simulation has been widely studied, particularly for clinical or emergency settings. However, the main part of these studies only includes HCSPs from up to three different professional backgrounds (Langton *et al.*, 2021). Despite its increasing importance in healthcare, IPC training for PHCUs that includes all relevant professions has not yet been established. In Austria, HCSPs are characterized by diverse levels of education (university and non-university programmes) and providers (universities, applied universities, vocational schools). Due to this separation of training institutions, little or no joint lectures or joint continuing educational programmes (for all professions in PHCUs) on IPC are currently available. Despite this fragmentation in education, HCSPs from these varied backgrounds are required to work together seamlessly in their daily practice. Building bridges to other professions within the training programmes and therein focusing on IPC can be beneficial to future HCSPs (Mette *et al.*, 2016; Rinnhofer *et al.*, 2022). Nevertheless, IPC simulations designed specifically for PHC settings remain less common (Lunde *et al.*, 2022; Waltz *et al.*, 2023), although they have been shown to improve communication skills, self-efficacy, and understanding of different professional roles, teamwork, and patient safety (Houzé-Cerfon *et al.*, 2019; Kyrkjebø *et al.*, 2006; Tofil *et al.*, 2014).

The integration of PHC in health and social care education offers a unique opportunity for interprofessional learning and partnerships between students, staff, clinicians, and service users (Mills *et al.*, 2020). Understanding the historical context and underlying values of health and social care is crucial to bridge the gap between services. Evaluating outcomes in health and social care partnerships is complex and requires a mix of context, history, evaluation methods, and theoretical approaches (Miller, 2017). The REALISE study employs a combination of questionnaires via online surveys (including ICCAS and ISVS) and observations (TAS) to evaluate its main outcomes. The ICCAS, which is used for primary results at T1 of the REALISE study, was shown to be a useful and valid instrument (Archibald *et al.*, 2014; Violato and King, 2019). The ISVS is reliable and suitable for research activities on IPC with healthcare students as well as HCSPs, and its two short nine-item equivalent forms are, due to their agreement, suitable to assess change resulting to IPC, which makes it a suitable instrument to use in the study at hand (King *et al.*, 2016; Mahler *et al.*, 2023). The measurement of teamwork is of high importance in this study setting; for this reason, the TAS is integrated, which showed valid psychometric properties for the assessment of teamwork in medical training (Kiesewetter and Fischer, 2015).

Sustainability in healthcare and social education not only aligns with global trends in environmental consciousness but also prepares professionals to address the health challenges posed by climate change and resource limitations (Brennan and Madden, 2023). Universities play a significant role in delivering health and social care education at different levels, promoting social responsibility and lifelong learning through sustainability (Mills *et al.*, 2020). These integrated approaches aim to address policy and practice challenges that have existed in different countries for decades (Miller, 2017). The protocol of the REALISE study contributes to the development of a didactic concept that integrates IPC and sustainability into education and training for HCSPs. The next steps involve refining this concept for broader implementation, both as a fixed element in basic education and in continuing professional development. To optimize the effectiveness of such training, careful consideration must be given to group composition, ensuring that heterogeneity and variability of professions enrich the learning experience (Ntsiea *et al.*, 2021). The significant differences in student numbers across professions require thoughtful planning, including the alignment of timetables and curricula to facilitate interprofessional education across different institutions. The results of the REALISE study will therefore provide a fundamental basis for collaborative implementation.

## Conclusion

This paper offers insights for the evaluation of an innovative didactic concept for IPC within the PHC setting. By enhancing IPC principles with sustainability-focused education, diverse simulation methods, and e-learning components, this approach aims to improve interprofessional competencies and address the evolving demands of primary care on HCSPs. The study’s findings will contribute to the development of evidence-based training strategies, supporting the effective implementation of interprofessional education in both basic and continuing education programmes for HCSPs.

## Data Availability

Further details of the study protocol presented in this paper are available on reasonable request from the corresponding author after publishing all data.
